# Expanding the Understanding of Content of End-of-Life Dreams and Visions: A Consensual Qualitative Research Analysis

**DOI:** 10.1089/pmr.2020.0037

**Published:** 2020-07-07

**Authors:** Rachel M. Depner, Pei C. Grant, David J. Byrwa, Sarah M. LaFever, Christopher W. Kerr, Kelly E. Tenzek, Susan LaValley, Debra L. Luczkiewicz, Scott T. Wright, Kathryn Levy, MSW AdvStat

**Affiliations:** ^1^ Department of Research, Hospice and Palliative Care Buffalo, Cheektowaga, New York, USA.; ^2^ Department of Counseling, School and Educational Psychology, Department of Family Medicine, University at Buffalo, The State University of New York, Buffalo, New York, USA.; ^3^ Jacobs School of Medicine and Biomedical Sciences, Department of Family Medicine, University at Buffalo, The State University of New York, Buffalo, New York, USA.; ^5^ Department of Communication, Department of Family Medicine, University at Buffalo, The State University of New York, Buffalo, New York, USA.; ^6^ Primary Care Research Institute, Department of Family Medicine, University at Buffalo, The State University of New York, Buffalo, New York, USA.; ^4^ Behavioral VA Health Care Line (BVAC), VA Western New York Healthcare System, Buffalo, New York, USA.; ^7^ University Counseling Center, University of Rochester, Rochester New York, USA.; ^8^ Department of Planning and Research, Trocaire College, Buffalo, New York, USA.

**Keywords:** consensual qualitative research, end-of-life dreams and visions, hospice care

## Abstract

***Background:*** Research has established End-of-Life Dreams and Visions (ELDVs) as prevalent, meaningful valid experiences that may help patients cope with illness and approaching death. However, no inductive qualitative analysis has explored the phenomenology of ELDVs from the perspective of hospice homecare patients.

***Objective:*** The purpose of this study is to evaluate the content of ELDVs by using a rigorous qualitative approach.

***Design:*** Five hundred forty-eight ELDVs were collected from weekly interviews of hospice homecare patients and analyzed by using Consensual Qualitative Research Methodology.

***Settings/Subject:*** Participants were enrolled in a county-wide hospice homecare program between January 2013–March 2015.

***Results:*** The following domains emerged: (1) Interpersonal, (2) Affective Experience and Reflection, (3) Activities, and (4) Setting/Location.

***Conclusions:*** This study suggests that ELDV content may include a broader spectrum of experiences that reflect waking life than previously believed. Clinical implications suggest that it may be important for providers to engage with ELDVs, as they are psychologically significant experiences that may be a source of clinical insight.

## Introduction

Research has shown that subjective experiences such as end-of-life dreams and visions (ELDVs) play an important role in the dying trajectory by offering comfort, psychological/spiritual solace, and meaning-making.^1–15^ The ELDVs involve mental and sensory activity while the patient is asleep (dreams) or awake (visions) and are typically reported to include seeing or feeling the presence of deceased loved ones. Research has evolved from case-based anecdotes and caregiver second-hand accounts^4,5,9,11,13–17^ to reports of patient perspectives.^1,2,10^ More recently, the scope of ELDV research has expanded to explore the impact on post-traumatic growth of patients^6^ and bereavement outcomes of familial caregivers.^18^

The ELDVs have often been dismissed by medical professionals as alterations in cognition (e.g., delirium). Although the dying process is frequently complicated by delirium,^19–21^ there are key elements that distinguish ELDVs from this type of cognition (Table 1). Delirium includes a breakdown in attention and awareness,^22^ whereas individuals experiencing ELDVs typically demonstrate organized thoughts.^1,10^ This distinction is clinically important, as medical professionals can play an important role facilitating meaning and comfort by not unnecessarily medicating patients with ELDVs. Professionals' failure to understand this distinction may even prevent the patient from engaging in a meaningful experience that is intrinsic to the dying process.

**Table 1.** Conceptualization and Differentiation of End-of-Life Dreams and Visions and Delirium

Sphere of being	Delirium	ELDV
Physical	Agitated movements, picking	Mild at rest
Cognitive	Disorganized, confused	Heightened acuity and recall
Relational	Withdrawn, disconnected	Engaged, connected
Emotional	Distressed	Comforted, calm
Mental	Sense of disbelief	Sense of reality
Existential/spiritual	Disconnected from spiritualty	Peaceful, transcendent

This table demonstrates the multifaceted clinical differences between the phenomenon of ELDVs and the experience of delirium; it is meant to be used as an educational but not diagnostic tool.

ELDVs, end-of-life dreams and visions.

The ELDVs may serve as a psychological mechanism that helps patients process and/or cope with approaching death.^7,23^ Although typically meaningful and calming, ELDVs can be potentially distressful,^2,24^ potentially even more for those with a history of trauma, comorbid mental health disorders, and/or increased existential suffering. Dream experiences may mirror waking life experiences in healthy^25,26^ and chronically ill/dying individuals,^7,23,25–27^ and they can be used as a nonthreatening avenue for engaging in difficult conversations about death/dying or past experiences. Although one study has explored how the interpretation of ELDV content might facilitate meaning-making at the end of life,^7^ much remains unknown about the phenomenological experience of ELDVs. A more thorough understanding of ELDV content might inform further development of psycho-social-spiritual treatment for dying patients. Therefore, the goal of this study was to evaluate the content of ELDVs as reported by patients enrolled in hospice homecare by using consensual qualitative research (CQR) methodology.

## Methods

This is a qualitative analysis of 548 ELDV reports from 55 people enrolled in a hospice homecare program. The study was approved by the Social and Behavioral Research Institutional Research Board of a midsize public university in New York (FWA00008824) on 11/14/2012.

### Participants

#### Patients

Participants were enrolled in a county-wide hospice homecare program between January 2013 and March 2015. Researcher visits occurred in patient residences. All participants were (1) currently enrolled in a hospice homecare program, (2) older than the age of 18, (3) able to provide consent, and (4) had a Palliative Performance Scale (PPS)^28^ score of at least 30%. Patients were not eligible for this study if they had diagnoses affecting cognition or if there was a language barrier.

### Measures

#### Demographics

Participants reported age, gender, race, marital status, and religious affiliation. The PPS and additional relevant diagnostic data were collected from electronic medical records (EMRs).

#### Cognitive and health status

Clinical information was collected and reviewed before each study visit. The confusion assessment method (CAM), a validated clinical tool that assesses delirium,^21,29^ was administered to assess appropriateness for ongoing participation.

#### Semi-structured dream interview

A semi-structured interview was developed for this study based on previous research^1,2^ and clinical experience. Interviews were only administered after participants reported at least one ELDV (Table 2).

**Table 2.** Structured Dream Interview Questions

Question
Please tell me about your dream/vision.
Who or what are you seeing in these dreams or visions?
What were the people (or figures, animals) in your dreams (or visions) doing?
How did you feel about this dream or vision? Or what was your reaction to this dream or vision?
How do these dreams or visions impact your sense of meaning and purpose of your life?
How do these dreams or visions impact your understanding of your relationships?
Do these dreams or visions affect the way you see death and dying? If so, how?
Does this dream or vision affect any unresolved issues in your life?
Were you or the people (or figures, animals) in your visions (or dreams) going somewhere or preparing to go somewhere?

This table includes all structured questions utilized in the interview process but does not include unique follow-up probes. These questions are derived from previous research^1,2^ and clinical experience.

### Procedures

Participants were recruited based on clinician referral or review of EMRs. Individuals meeting criteria were given the opportunity to participate regardless of whether they reported ELDVs or not. Verbal consent was reobtained at each visit. Participants were not provided any additional benefits or compensation. All participants were given a notebook to optionally record ELDVs. Weekly data collection was completed by a researcher or clinician from the time of consent until participants were unable to/chose not to continue. At the start of each weekly visit, the CAM was administered to ensure that the participant was not delirious. All data from semi-structured interviews were transcribed verbatim and de-identified.

### Data analysis

The CQR methodology was used to analyze data.^30,31^ This analysis incorporates elements of both constructivism and post-positivism as the primary research paradigm by using an inductive, systematic qualitative analysis that adopts a team consensus approach. A core team and external auditors engage in all steps of the process. The process includes (1) sorting data into domains or overarching themes, (2) converting raw data into core ideas, and (3) cross-analysis, which evaluates within each domain for emergent categories (or subthemes). Representativeness across all categories is calculated. Representativeness was calculated per CQR guidelines by the number of participants who described at least one ELDV within that category. All/all but one participant = general representativeness, between half and all = typical, more than four but less than half = variant, and less than three participants = rare.

In this study, four researchers (R.M.D., P.C.G., D.J.B., S.M.L.) comprised the core data analysis team. Personal biases were recorded, discussed, and reported in Supplementary Data. One auditor (K.E.T.) reviewed the work of the core team. At each step of the CQR process, team members individually reviewed the data, then reviewed as a team, and finally reached an agreement before moving on to the next step. At each stage, the external auditor reviewed and provided feedback, which is incorporated at the discretion of the core team.

To limit bias, data were randomized so that proximity to death relative to reported ELDV was unknown. On completion of analysis, data were reordered based on days before death and categories and subcategories were analyzed for frequency.

## Results

### Demographics

Eighty-three patients participated in 842 weekly visits, resulting in 548 ELDV reports from 55 participants (66%). Most participants were aged 61 to 101 years (89%), white/European (97%), female (70%), Christian (82%), and with a primary diagnosis of cancer (47%). The modal number of days of participation in the study was 14, but it ranged from 5 to 206 (mean 145 days; SD 123) (Table 3).

**Table 3.** Participant Demographic Data

Characteristic	Group	N	%
	Total	55	100
Age	18–60	6	11
	61–101	49	89
Gender	Female	39	70
	Male	16	30
Race	White/European	53	97
	African American	2	3
Diagnosis	Cancer	26	47
	COPD	17	31
	CHF	7	13
	Other	5	9
Religious affiliation	Christian	45	82
	Atheist/none affiliated	7	13
	Jewish	2	4
	Other	1	1

This table displays the participant's self-reported demographic information, including age, gender, race, diagnosis, and religious affiliation.

CHF, congestive heart failure; COPD, chronic obstructive pulmonary disease.

### CQR results

Four domains emerged from the dataset: *Interpersonal*, *Affective Experience and Reflection*, *Activities*, and *Setting/Location*.

#### Interpersonal domain

The interpersonal domain was operationalized as an ELDV description featuring people/animals, and information about interactions. Within this domain two primary categories emerged (1) Characters and (2) Relational Interactions (Table 4).

**Table 4.** Interpersonal Domain Categories and Subcategories with Case Representation and Frequency

Category/subcategory	Frequency
1. Characters	General
Self-present in dream/vision	General
Family	Typical
Parents/parental figures	Variant
Siblings	Variant
Spouse/partner	Variant
Other relatives/extended family	Variant
Domestic pets	Variant
Unfamiliar/unknown	Typical
Miscellaneous characters	Typical
Peer relationship	Variant
Crowd/group	Variant
High profile/authority figures	Variant
Atypical/incongruent character descriptions	Variant
Characters who are alive again	Variant
Baby/child	Variant
Wildlife/unknown animals	Variant
2. Relational interactions	General
Close connection	Typical
Neutral connection	Typical
Alone	Variant
Disconnected/unable to connect	Variant
Moving toward	Variant
Conflictual connection	Variant
Moving away	Variant

This table reports the categories and subcategories from the interpersonal domain as well as representativeness in the dataset. The frequency labels are determined by CQR (Hill)^30^ and are as follows: General, the category is represented in all or all but one case; typical, occurs between half and less than all; and Variant, equals less than half but more than three cases.

CQR, consensual qualitative research.

*Characters* emerged with *general* representativeness, and they were quite diverse with 11 subcategories. In *general*, participants identified themselves as present within the ELDV. *Typically*, participants reported ELDV experiences featuring *Family*. One participant shared, “I see my mother and she talks to me.”

It was also *typical* for ELDVs to include people who were *Unfamiliar/Unknown*. One participant reported, “Other people were standing around us but I did not recognize anyone else.” Likewise, participants *typically* reported *Miscellaneous Characters*, such as healthcare providers, “stereotypical New York guys,” people from TV commercials, and others. For example, “I remember Mrs. Peloquin, first person to be a weekly client. I did her hair for a very long time.”

The final seven subcategories describing characters all emerged with *variant* representativeness. *Peer Relationships* included friends, classmates, lovers, and co-workers and were distinguished by equivalent levels of power and social status. One participant shared, “I had a dream about a couple I used to be good friends with when my husband was alive.” This contrasts with *High Profile/Authority Figures*, including characters beyond/above the power level of the dreamer who would not usually be in the same social circle. *Atypical/Incongruent Character Descriptions* included people who participants reported knowing, but whose characteristics did not match how they knew them in waking life, such as loved ones who appeared to have different physical characteristics, ages, or health status. One participant described, “I hear my three children playing up above me like when they were kids. I almost yell out and tell them to get to bed but then I realize they are all grown up.” Similarly, participants reported experiencing ELDVs with *Characters Who are Alive Again*, including people who the participants know are already deceased. One said, “[I] dreamed about Mom and Dad; they were old in the dream but [are] deceased in life.” Comparatively, participants were equally likely to discuss the presence of a *baby/child*, *Groups/Crowds*, and *Wildlife/Unknown Animals*.

Another category that emerged with *general* representativeness was *Relational Interactions*, which included information about exchanges during the ELDV between the participant and others. It was *typical* for participants to report interactions as *Close Connections,* or any relational description that is intimate and/or emotionally close. This encompassed feeling a presence that was positive, a sense of closeness, and/or connecting through verbal and nonverbal displays. One participant described feeling the warm presence of her husband, “[I am] very happy, especially at night, when I wake up and feel like he was snuggled up against me.”

It was equally likely for participants to report ELDVs that included *Neutral Connections*. This interaction was differentiated as a lower level of relational engagement or not having a highly reactive tone, neither warm/intimate nor conflictual. For example, “My dad came to me in a dream and we were doing day-to-day things.”

Interestingly, a few subcategories emerged that focused on the lack of interaction or a thwarted interaction. Examples include, “I was alone on a train.” or “I am at a county fair with my parents and sister…I became annoyed with the family because they keep taking off for another area without letting me know.” Another relational dynamic was variant *Conflictual Connection*, which includes relationships described as aggressive and/or hostile and ranged from minor conflict/disagreement to full on physical/relational harm. One participant recalled, “Then he [dog] came up on the bed, looked right at me, smiled and peed. Then the last second of the dream was me chasing him through the house with a pot.”

#### Affective experience and reflection

This domain emerged with a wide range of categories and subcategories, including two categories with general representativeness (Table 5).

**Table 5.** Affective Experience Categories and Subcategories with Case Representation and Frequency

Category/subcategory	Frequency
1. Feelings/emotions	Typical
Peace, calm, comfort	Variant
Nice, good, great	Variant
Happiness, excitement, pleasure	Variant
Humor, silly, laughter	Variant
Curiosity, wondering	Variant
Neutral/no strong feelings, apathy	Variant
Disturbing, distressing, scary	Variant
Uncertainty, confused, puzzled	Variant
Anger, frustration, disappointment	Variant
Anxiety, stress, concern	Variant
Surprised, startled, shocked	Variant
Complex feelings	Variant
Sadness, sorrow, down	Variant
2. Reflections	Typical
Nostalgia	Typical
Meaning and Coherence	Typical
Dream Commentary	Typical
Felt odd, strange, weird	Variant
Real, vivid	Variant
Normal	Variant

This table reports the categories and subcategories from the Affective Experience domain as well as representativeness in the dataset. The frequency labels are determined by CQR (Hill)^30^ and are as follows: General, the category is represented in all or all but one case; typical, occurs between half and less than all; and Variant, equals less than half but more than three cases.

Participants *generally* described their dreams with *Feelings/Emotions*. This is grounded primarily in direct statements such as “It made me feel happy” or “now that I think about it I am sad.” Twelve main subcategories with variant representativeness emerged, including traditionally positive emotions such as: (1) peace/calm/comfort, (2) nice/good/great, (3) happiness/enjoyment/excitement/pleasure/fun, (4) humor/silly/laughter; and (5) curiosity/wondering. One participant exemplified this, saying “it was comforting to see the trees and it was a beautiful fall day, I felt happy.”

Equally, this category included emotions *typically* viewed as distressing: (1) uncertain/confused/puzzled, (2) disturbing/distressing/scary/fearful/upset, (3) mad/angry/frustrated/disappointed/irritated, (4) worried/anxious/stressed/overwhelmed/concerned, (5) surprised/startled/shocked, and (6) sadness/sorrow/blue. One participant reported a specific affective experience, “I was watching children play in my house and someone got hurt I was very upset by this. I woke up crying.”

Two final subcategories within Feelings/Emotions were *Neutral/No Strong Feelings* and *Complex Emotions*. The latter was defined as the presence of multiple feelings within one dream that may be at odds with or contrast one another, adding complexity to the ELDV. Examples included, “I was surprised how accurate it [the dream] was. It was so good but it was also deeply, deeply disturbing.”

The second category in the Affective domain is *Reflection*. Participants typically reflected on an element of *Nostalgia*, a longing to reconnect with a person, place, or experience from the past. One participant shared an ELDV of her friend who was deceased, “She is not interacting with me but I like having her there. When I wake up, I think, oh man, get back here!”

It was typical that participants reported at least one ELDV represented in the *Meaning and Coherence* category, in which they spontaneously engaged in sense-making. This category is exemplified by the following report, “…my dreams have increased and I think I'm working things out in my dreams or trying to come to terms with my sickness in my dream.” In addition, it was also typical that participants reported details or *Dream Commentary* about their ELDV experience. The remainder of subcategories in this section occurred with variant frequency.

#### Activities

This domain evaluates actions/events that occurred within the ELDV and resulted in 14 subcategories (Table 6). Typically, participants reported activities related to *Traveling and Movement*. One said, “I am on a bicycle coming down a steep curving mountain highway. I find that the speed of the bike is getting dangerously fast.”

**Table 6.** Activities Categories and Subcategories with Case Representation and Frequency

Category	Frequency
1. Traveling and movement	Typical
2. Talking/verbal communication	Typical
3. Observing and watching	Typical
4. Sport, play, and recreation	Variant
5. Harm and injury	Variant
6. Domicile/household chores	Variant
7. Anticipatory behaviors	Variant
8. Attempts	Typical
9. Acquiring/consuming	Variant
10. Work/school related	Variant
11. Destruction/rebuilding	Variant
12. Prosocial activities	Variant
13. Searching and pursuit	Variant
14. Positions of inactivity	Variant

This table reports the categories and subcategories from the Activities domain as well as representativeness in the dataset. The frequency labels are determined by CQR (Hill)^30^ and are as follows: General, the category is represented in all or all but one case; typical, occurs between half and less than all; and Variant, equals less than half but more than three cases.

Similarly, participants typically reported *Verbal Communication* and *Observing and Watching* as common. Interestingly, it was also typical for the ELDVs to involve an *Attempt* to do something, including working toward a specific goal. A participant shared, “I was going to my friend's cottage up a mountain, was snowing hard, had to walk back but never got anywhere.”

The remaining subcategories were represented with variant frequency and included (1) *Acquiring/Consuming Behaviors*, such as shopping, eating and drinking, getting a gift, and obtaining something; (2) *Anticipatory Behaviors*, including planning or preparing for something; (3) *Search and Pursuit*, looking for someone/something, being followed/chased; (4) *Harm and Injury*, which was associated with bodily harm, fighting, physical attack, violence to the body, shooting, or killing; and (5) *Position of Inactivity*, described as sitting still, lying down, sleeping, being stationary, and/or lack of activity.

#### Setting/location

The final domain includes the setting and backdrop of ELDVs. Individuals typically reported that the environment was *Familiar/Known* and involved the *Natural Environment*. Some examples included, “swimming and playing with a dolphin in the ocean,” and “I was in a creek…searching for a certain kind of rusty rocks.” Similarly, participants typically described settings related to *Transportation and Travel* (e.g., train stations, buses, planes, etc.).

Typically, people reported ELDVs set in their *Home/Residence*, and this included an overall home, structure, or building as well as objects or rooms associated with home, for example, kitchen. In addition, it was typical for participants to describe settings or locations with cues related to *Spatial Awareness and Directionality*, or where exactly the individual, other characters, and/or objects were within the setting, including references to top, bottom, up, down, side to side, inside/indoors, and outside/outdoors.

Finally, it was typical for individuals to describe settings related to *Institutions of Daily Life*. This included *Social Gatherings* such as weddings, picnics, and graduations, as well as subcategories such as *Places of Work*, *Places of Business*, *Places of Education*, *Places of Play* (e.g., pool, theatre, cottage, camp, etc.), and *Places of Worship*. For example, “I was sitting in a kiddie pool outside in the grass with my home health aide.” The remaining categories within this domain occurred with variant frequency (Table 7).

**Table 7.** Setting and Location Categories and Subcategories with Case Representation and Frequency

Category/subcategory	Frequency
1. Transportation and travels	Typical
2. Natural environment	Typical
3. Familiarity of location	Typical
Known/familiar	Typical
Unknown/unfamiliar	Variant
4. Spatial awareness and directionality	Typical
5. Institutions of daily life	Typical
Social gatherings	Variant
Places of work	Variant
Places of business	Variant
Places of education	Variant
Places of play	Variant
Places of worship	Variant
Places of education	Variant
6. Residence/home	Typical
Kitchen	Variant
Bedroom/bed	Variant
House/home	Variant
MISC house stuff	Variant
7. Settings associated with death and/or illness	Variant

This table reports the categories and subcategories from the Setting and Location domain as well as representativeness in the dataset. The frequency labels are determined by CQR (Hill)^30^ and are as follows: General, the category is represented in all or all but one case; typical, occurs between half and less than all; and variant, equals less than half but more than three cases.

## Discussion

This study explored the unique experiences of ELDVs as reported by people receiving hospice homecare. Although some of the findings are congruent with previous research (e.g., comforting ELDVs and activities connecting with family/loved-ones),^1,2^ the results of this analysis showcase a much more expansive and diverse range of interactions, emotions, and activities. This may be due to the use of open-ended questions regarding dream content rather than checklists as in previous studies.^2,10^ Findings align with the conclusions of Fenwick and colleagues, who found these experiences “to be far broader than the archetypal image of ‘take-away’ apparitions or visions at the end of the bed.”^9^ This is also supported by other research that suggests that dreams may mirror waking life experiences and concerns of healthy and chronically ill and dying people.^8,17,23,25–27^

The majority of previous ELDV research conceptualizations have focused on deceased loved ones. In this study, participants reported deceased loved ones with equal representativeness as *Young people/Babies*. Only one other study reported participants experiencing children in ELDVs.^11^ Surprisingly, participants were also equally likely to report an ELDV that included *Family*, *Unknown/Unfamiliar People*, and *Miscellaneous Characters*. These findings broaden previous conceptualizations and suggest that people being featured in ELDVs may be more varied and complex, thus supporting the need to delve further into these experiences.

Participants described interactions within ELDVs that demonstrated *Close Connections* and *Neutral Connections* with equal frequency, where previous literature has suggested that the majority of interactions are characterized as close and/or comforting.^1,2,9,11^ It is noteworthy that participants also reported conflictual interactions. This significantly expands the understanding of interpersonal dynamics in ELDVs to bear a closer resemblance to waking life. Likewise, the wide range of emotions reported indicates that ELDVs are more varied than previously believed.

Past research documented both the clarity and detail of ELDV experiences.^1,11–13^ The rich descriptions of activities and settings in ELDVs reported here go beyond previous accounts with content that is diverse and broad, and that is reflective of individual perceptions of disease progression, existential distress, and/or life review. As suggested in an earlier study, ELDVs can be an agent for positive psychological growth,^32^ and to assume a distressing/negative ELDV to be less meaningful or inconsequential is an oversimplification.

Understanding the phenomenological experiences of people at the end of life (EOL) is essential to the comprehensive treatment inherent in palliative and hospice care. When appropriate, clinical and interdisciplinary staff can explore ELDVs with patients and their families to gain understanding and build connections. Such experiences may not be universally easy to discuss, yet they present an opportunity to address important aspects of the person's life and dying experience.

Clinical significance has been noted by others who recognized the stigma felt by both the individual and clinicians around the topic of ELDVs.^33^ The importance of addressing ELDVs within a clinical framework is indicated given the prevalence^1,10,16,18^ and potential to provide comfort and closure.^1,3,6,15,32^ It is imperative for clinical staff to build efficacy on how to engage in discussion and validate ELDVs to reduce the stigma around this common EOL experience.

### Limitations

This study primarily included elderly, white, Christian females. The ELDV content of this group may not be generalizable to all and future research should explore ELDVs from other cultural, demographic, and environmental contexts. Second, only some participants recorded their ELDVs between weekly interviews and therefore descriptions may be subject to recall bias. Third, there was no relationship identified between the timing of interviews in relation to patient death and the content of the ELDVs contrary to previous research.^1^ This discrepancy may be related to the setting where data were collected (homecare vs. inpatient) or differences in interview protocols and frequency of interviews. Lastly, this study did not specifically collect any data with regards to history of trauma or mental illness. Future studies should more closely focus on the relationship between trauma, mental illness, and existential suffering and how it plays a role in ELDVs.

## Conclusion

The ELDVs are complex subjective experiences with the potential to provide comfort to the dying and insight to clinicians and families. They are valid, meaningful, and encompass a broader range of content and affect than what has been previously reported. Prior research has suggested that peace/comfort can be attributed to “seeing” deceased loved ones and feeling a warm presence. An alternative explanation may be that comfort is a product of making sense/meaning out of the ELDV and/or the ability to safely share the experience with others. Most importantly, it is critical to foster a sense of openness, awareness, and engagement around ELDVs when interacting with those nearing EOL.

## Supplementary Material

Supplemental data

## Abbreviations Used

CAM: confusion assessment method
CQR: consensual qualitative research
ELDV: end-of-life dream and vision
EMRs: electronic medical records
EOL: end of life
PPS: palliative performance scale
