# Supplementary material for: Expanding the Understanding of Content of End-of-Life Dreams and Visions: A Consensual Qualitative Research Analysis
Source: Palliat Med Rep. 2020 Jul 7;1(1):103–10. doi: 10.1089/pmr.2020.0037 (PMC8241341; doi:10.1089/pmr.2020.0037)
Supplement: Supplemental data [file Supp_Data.pdf]

Supplementary Data

Biases and Expectations

Researchers

R.M.D. is a 32-year-old European American female researcher and Counseling Psychology PhD candidate; has experience within the hospice setting, intermediate experience conducting consensual qualitative research (CQR); and completed 52% of the interviews. P.C.G. is a 37-year-old Asian Canadian female researcher with a PhD in Biochemistry; has experience within the hospice setting, intermediate experience using CQR; and completed 1% of the interviews. D.J.B. is a 26-year-old European American male medical student, with a master's degree in Nutrition Science, experience within the hospice setting, moderate experience conducting CQR; and completed 5% of the interviews. S.L. is a 38-year-old European American female researcher with a PhD in Community Health and Health Behavior with experience within the hospice setting, conducting qualitative research; has training in CQR; and did not conduct any of the interviews.

Auditor

K.E.T. is a 35-year-old European American female researcher with a PhD in Communication, expertise in health and interpersonal communication within the context of end of life, and extensive qualitative research methodology. This was their first experience with CQR as an auditor.
